# The Simplified Human Intestinal Microbiota (SIHUMIx) Shows High Structural and Functional Resistance against Changing Transit Times in *In Vitro* Bioreactors

**DOI:** 10.3390/microorganisms7120641

**Published:** 2019-12-03

**Authors:** Stephanie Serena Schäpe, Jannike Lea Krause, Beatrice Engelmann, Katarina Fritz-Wallace, Florian Schattenberg, Zishu Liu, Susann Müller, Nico Jehmlich, Ulrike Rolle-Kampczyk, Gunda Herberth, Martin von Bergen

**Affiliations:** 1 Department of Molecular Systems Biology, Helmholtz-Centre for Environmental Research―UFZ GmbH, 04316 Leipzig, Germany; stephanie.schaepe@ufz.de (S.S.S.); beatrice.engelmann@ufz.de (B.E.); katarina.fritz@ufz.de (K.F.-W.); nico.jehmlich@ufz.de (N.J.); ulrike.rolle-kampczyk@ufz.de (U.R.-K.); 2 Department of Environmental Immunology, Helmholtz-Centre for Environmental Research―UFZ GmbH, 04316 Leipzig, Germany; jannike-lea.krause@ufz.de (J.L.K.); gunda.herberth@ufz.de (G.H.); 3 Department of Environmental Microbiology, Helmholtz-Centre for Environmental Research―UFZ GmbH, 04316 Leipzig, Germany; florian.schattenberg@ufz.de (F.S.); zishu.liu@ufz.de (Z.L.); susann.mueller@ufz.de (S.M.); 4 Institute of Biochemistry, Faculty of Biosciences, Pharmacy and Psychology, University of Leipzig, 04103 Leipzig, Germany

**Keywords:** *In vitro* model, microbial community, flow cytometry, metaproteomics, metabolomics, short-chain fatty acids, intestinal microbiota, SIHUMIx, bioreactor

## Abstract

Many functions in host–microbiota interactions are potentially influenced by intestinal transit times, but little is known about the effects of altered transition times on the composition and functionality of gut microbiota. To analyze these effects, we cultivated the model community SIHUMIx in bioreactors in order to determine the effects of varying transit times (TT) on the community structure and function. After five days of continuous cultivation, we investigated the influence of different medium TT of 12 h, 24 h, and 48 h. For profiling the microbial community, we applied flow cytometric fingerprinting and revealed changes in the community structure of SIHUMIx during the change of TT, which were not associated with changes in species abundances. For pinpointing metabolic alterations, we applied metaproteomics and metabolomics and found, along with shortening the TT, a slight decrease in glycan biosynthesis, carbohydrate, and amino acid metabolism and, furthermore, a reduction in butyrate, methyl butyrate, isobutyrate, valerate, and isovalerate concentrations. Specifically, *B. thetaiotaomicron* was identified to be affected in terms of butyrate metabolism. However, communities could recover to the original state afterward. This study shows that SIHUMIx showed high structural stability when TT changed—even four-fold. Resistance values remained high, which suggests that TTs did not interfere with the structure of the community to a certain degree.

## 1. Introduction

The human intestine harbors hundreds of bacterial species that are associated with human health and disease [1,2]. This association is mainly due to changes in metabolic interactions with the host caused by changes in the bacterial community structure and function. One of the primary roles of intestinal microbiota is the conversion of nutrients into bioactive compounds, which are taken up by the host [3,4]. Due to the enzymatic break-down of carbohydrates, proteins, and fatty acids, the intestinal microbiota produces essential nutrients, such as short-chain fatty acids (SCFA) and vitamins [5]. SCFA provide energy for intestinal epithelial cells and modulate the host immune system and, therefore, play a beneficial role in the host’s health [6]. While carbohydrate fermentation results in SCFA production, the fermentation of proteins and amino acids results in the production of branched short-chain fatty acids (BCFA). BCFA such as isovalerate, isobutyrate, and 2-methyl butyrate are produced due to the fermentation of valine, leucine, and isoleucine and serve as precursors for fatty acid synthesis or as nitrogen donors for the production of other amino acids [7,8]. The concentration of these bioactive compounds depends on daily food intake and the type of food. Hence, nutrient concentrations in the gut change and can, thereby, affect metabolic interactions. Changes in nutrient concentrations can not only vary due to different amounts of food intake but also due to variations in intestinal transit times, dietary amount, and health state (e.g., infections) [9].

In the human population, differences in intestinal transit times (TTs) are often observed. They have been associated with differences in stool frequency and, therefore, have been linked to changes in the gut microbiota composition [10,11]. In healthy human individuals, the whole gut TT varies but takes approximately 27 h from which 18 h refer to the colonic TT [12,13,14]. Child et al. compared 20 h and 60 hTTs and found that 20 hTT resulted in a decrease or loss of bacterial populations, e.g., *Rumminococcus* and *Roseburia* compared to 60 hTT [15]. In addition to the community composition, the metabolism was also affected. Tottey et al. observed, at an increased TT from 48 h to 96 h, a decrease in biomass and an increase in protein fermentation, while SCFA production remained mainly unaffected [16]. Furthermore, changes in TT also affected the fermentation production of a single species. For example, *C. tyrobutyricum* showed a higher butyrate production within a shorter TT of 8 h compared to 16.7 h [17]. Nevertheless, all of these intestinal *in vitro* models used complex fecal communities, which evolve differently in *in vitro* systems [18,19].

The effect of intestinal TTs was investigated in the past in various bioreactor models by the use of different TTs, but effects on structure and function have not been fully understood [15,16,20]. *In vitro* bioreactor systems are useful tools to investigate environmental stressors on microbial communities, since they overcome the limitations of conventional culture techniques [5]. Most *in vitro* bioreactor models use fecal inocula and, therefore, face the problem of missing the establishment of a reproducible complex microbial community [21]. Moreover, for metaproteome analysis of complex microbial communities, the major challenge is to achieve sufficient proteome coverage in order to generate a comprehensive picture of the community structure and function [22,23]. To overcome these challenges, we recently established an extended simplified human intestinal microbiota (SIHUMIx) for *in vitro* use (Krause et al., in revision at *Gut Microbes journal*). This model community consists of eight bacterial species resembling, to a large extent, the metabolic activities found in the human intestine [24]. Rothe et al. [25] also selected the strains because the genome sequences were available. Functions that are known to be fulfilled by each species are given in Supplementary Material Table S1. Several previous studies with SIHUMIx have been published in which gnotobiotic mice were seeded with SIHUMIx or part of the SIHUMIx strains to investigate an interaction between host and gut microbiota [26,27,28,29]. Data from Becker et al. were also used for modelling approaches [30]. We found that SIHUMIx maintained its structure to a high degree when cultivated continuously and reached the ability to stay essentially unchanged after five days, which serves as the starting point for experimental treatments (Krause et al., in revision at *Gut Microbes journal*). In contrast to complex microbial communities, it is easier to unravel the impact of environmental stressors on a structural and functional level in simplified microbiota [5]. However, low complex bacterial communities are expected to be less stable against perturbations compared to complex communities [11].

Since different intestinal TTs have been shown to affect complex bacterial communities, the effects on structure and function are not fully understood. Therefore, we cultivated SIHUMIx with a 24-hTT until the communities adapted to the system. The adaptation was followed by changes in TT to a faster (12 h) and a slower value (48 h), which results in a high, medium, and low availability of nutrients. The community structure and activity of SIHUMIx were investigated using microbial flow cytometry, metaproteomics, and short-chain fatty acids (SCFA) metabolomics analysis. We aimed to investigate, if (1) SIHUMIx shows structural or functional changes in response to a shift in TT, if (2) these parameters recover from lower and higher TTs to the original state, and if (3) visualized changes are restricted to specific species.

## 2. Materials and Methods 

### 2.1. Simplified Human Intestinal Microbiota—SIHUMIx

The extended simplified human intestinal microbiota (SIHUMIx) consist of the following eight bacterial strains: *Anaerostipes caccae* (DSMZ 14662), *Bacteroides thetaiotaomicron* (DSMZ 2079), *Bifidobacterium longum* (NCC 2705), *Blautia producta* (DSMZ 2950), *Clostridium butyricum* (DSMZ 10702), *Clostridium ramosum* (DSMZ 1402), *Escherichia coli* K-12 *(MG1655)*, and *Lactobacillus plantarum* (DSMZ 20174) [24]. Further information on functions fulfilled by SIHUMIx is provided (Table S1). The cultivation protocol, growth conditions, and medium ingredients are provided in Supplementary Material S2.

### 2.2. Experimental Set-Up

For inoculation of the bioreactor system, the single strain bacteria were thawed from a fresh glycerol stock two weeks before the experiment started and grown in Brain-Heart-Infusion (BHI), as described (Supplementary Material Table S2). Bacteria from three-day-old cultures were counted at the Multi-Sizer 3 (Beckman Coulter, Brea, United States) and used for inoculation. On the day of inoculation (d0) 1 × 10^9^ bacteria per strain (a total of 8 × 10^9^ bacteria per 250 mL) were inoculated into the bioreactor. The continuous cultivation started after 24 h.

**Figure 1:**
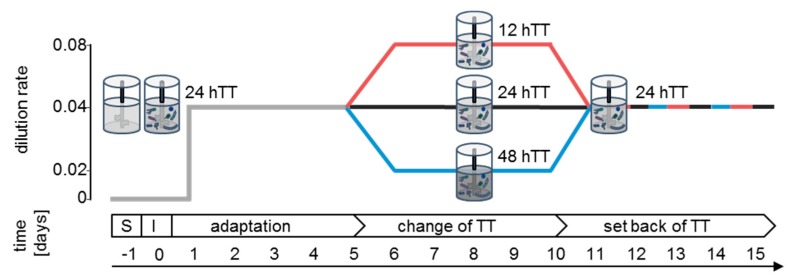
Set up of the bioreactor experiment: six bioreactors were run sterile for 24 h and inoculated with SIHUMIx (1 × 10^9^ cells per species per 250 mL) on day zero. On day one, medium pumps were set to a dilution rate of 0.04 h^−1^ until day five (adaptation). On day five, the dilution rate was changed to 0.02 h^−1^ (48 hTTI and II) or 0.08 h^−1^ (12 hTTI and II) in two bioreactors, respectively (change of TT), and set back to 0.04 h^−1^ on day 10 to day 15 (set back of TT). By setting the dilution rate to 0.08 h^−1^/0.04 h^−1^/0.02 h^−1^, the medium in the bioreactor was fully exchanged, which resulted in TTs of 12 h, 24 h, and 48 h, respectively. S = sterile run. I = inoculation.

The bioreactor run can be divided into three phases: (i) the adaptation phase where the bacterial community was established (d1–d5), (ii) the intermediary phase where the effect of different nutrient flowrates on the community was investigated (d6–d10), and (iii) the last phase in which the varying TTs were set back and the communities were rebalanced (d11–d15). The control bioreactors (labeled as 24 hTTI and II) were run with a dilution rate of 0.04 h^−1^ (24 h transit time, hTT) during the whole experiment (d1–d15). The dilution rate was calculated as reported in Macfarlane et al. [20], which is equal to the physiological TT of the human colon [13]. In four other bioreactors, dilution rates of 0.04 h^−1^ were maintained for the adaptation phase and the set-back phase and were set to a dilution rate of 0.08 h^−1^ (labeled as 12 hTTI and II) and 0.02 h^−1^ (labeled as 48 hTTI and II), respectively, within the intermediary phase (d6–d10) (Figure 1).

### 2.3. Sampling and Analysis

During the whole experiment, samples were taken every 24 h starting the day after inoculation (d1). The stability properties of the community structure were analyzed using microbial flow cytometry [31,32]. Cell numbers were determined with electrical sensing using a Multisizer 3 Coulter Counter (Beckman Coulter, Brea, United States). The community structure and the functionality of SIHUMIx were analyzed using meta-proteomics. After sampling, the bacterial suspensions were centrifuged at 3200× *g* for 10 min at 4 °C and immediately frozen at −80 °C for subsequent sample analysis or used for the fixation of microbial flow cytometry or dry weight assessment. Supernatants of bacterial pellets were used for functional analysis with targeted metabolomics to perform SCFA profiling [33].

### 2.4. Microbial Flow Cytometry

#### 2.4.1. Sample Preparation

The bacterial suspension was centrifuged (3200× *g*, 10 min, 4 °C) in conic glass tubes. Bacteria were treated with 2% formaldehyde [stock: 8% formaldehyde pH 7, diluted with PBS (6 mM Na_2_HPO_4_, 1.8 mM NaH_2_PO_4_, and 145 mM NaCl with bi-distilled water, pH 7)] for 30 min. The bacteria were centrifuged and resuspended in 70% ethanol for further fixation and long-term storage at −20 °C.

After a minimum of one day at −20 °C, the samples were stained with 0.24 µM 4′,6-di-amidino-2-phenyl-indole (DAPI, Sigma-Aldrich, St-Louis, USA) overnight, according to Koch et al. [31]. The measurement was performed according to Gelder et al. [34], but with a different neutral density filter (ND 2.6) for the side scatter (SSC) and measuring 250,000 cells in the cell gate (Supplementary Material, Figure S1). Raw cytometric data can be found at www.flowrespository.org with the flow repository ID: FR-FCM-Z24C.

For flow cytometric analysis and statistical data analysis, FlowJo V10 (FlowJo, LLC, Ashland, USA) was used to visualize each sample in 2D plots using forward scatter (FSC) vs. DAPI fluorescence. The relative cell abundance per gate was exported as .txt and jointly evaluated in R (vegan package) [35]. 

#### 2.4.2. Calculation of Stability Properties

The stability properties (i.e., constancy, resistance, and recovery) of SIHUMIx were quantified on the basis of cytometric data. For each bioreactor, constancy was interpreted by a constancy space, which is a multi-dimensional dissimilarity space where the radius is determined by the variation of chosen community states (deviation values determined by Canberra distance, CD). The constancy space was defined using samples from the two 24 hTTI and II using community states from the end of the adaptation phase (day 5) to the end of the experiment (day 15, *n* = 11), which resembles intrinsic community variation over time. The larger radius of the two constancy spaces from the two control bioreactors (i.e., 24 hTTI, tr = 0.1447, Supplementary Material Figure S2) was used as the threshold value to indicate the highest possible community deviation without TT changes.

Additionally, stability properties were calculated by resistance values (RS) (i.e., the ability of a community to stay unchanged) and recovery values (i.e., the ability of a community to return into the constancy space). For that, the community states at the end of the respective adaptation phases (reference state, SRef, day 5 per bioreactor) were successively compared with the community states during the following days of cultivation. The resulting CD values were used to calculate the resistant behavior and recovery strength of the microbial community following published guidelines [32]. 

### 2.5. Metaproteomics

#### 2.5.1. Protein Extraction

An amount of 2 mL bioreactor liquid was taken, centrifuged (3200× *g*, 10 min, 4 °C), and the pellet was dissolved in 1 mL lysis buffer (10 mM Tris-HCl, NaCl 2 mg/mL, 1 mM PMSF, 4 mg/mL SDS). Bacteria were disrupted by bead beating (FastPrep-24, MP Biomedicals, Sanra Ana, CA, USA, 5.5 ms, 1 min, 3 cycles) followed by 15 min at 60 °C (Thermomixer comfort 5355, Eppendorf, Hamburg, Germany) and ultra-sonication (UP50H, Hielscher, Teltow, Germany, cycle 0.5, amplitude 60%). Protein concentration was determined with the bicinchoninic acid assay, according to the user manual (Pierce™ BCA Protein Assay Kit, Thermo Fischer Scientific, Waltham, MA, USA). Furthermore, 100 µg of protein was precipitated overnight at −20 °C with ice-cold acetone 1:5 (v/v) and centrifuged for 10 min at 14,000× *g*. The pellet was used for sodium dodecyl sulfate poly acryl amid one-dimensional gel electrophorese (SDS–PAGE). SDS-PAGE analysis, in-gel digestion, and protein purification with ZipTip® treatment were performed [36].

#### 2.5.2. Liquid chromatography mass spectrometry (LC-MS/MS) Measurement

An amount of 5 µg peptide lysate was injected into nanoHPLC (UltiMate 3000 RSLCnano, Dionex, Thermo Fisher Scientific, Waltham, MA, USA). Peptide separation was performed on a C18-reverse-phase trapping column (C18 PepMap100, 300 µm × 5 mm, particle size 5 µm, nano viper, Thermo Fischer Scientific, Waltham, MA, USA), which was followed by a C18-reverse-phase analytical column (Acclaim PepMap^®^ 100, 75 µm × 25 cm, particle size 3 µm, nanoViper, Thermo Fischer Scientific). Mass spectrometric analysis of peptides was performed on a Q Exactive HF mass spectrometer (Thermo Fisher Scientific, Waltham, MA, USA) coupled with a TriVersa NanoMate (Advion, Ltd., Harlow, UK) source in the liquid chromatography (LC) chip coupling mode. LC gradient, ionization mode, and the mass spectrometry mode were described [37].

#### 2.5.3. Data Analysis

Raw data were processed with Proteome Discoverer (v 2.2, Thermo Fischer Scientific, Waltham, MA, USA). The search settings for the Sequest HT search engine were set to Trypsin (Full), Max. Missed Cleavage: 2, precursor mass tolerance: 10 ppm, fragment mass tolerance: 0.02 Da. The protein-coding sequences of the eight SIHUMIx strains were downloaded from UniProt (Available online: http://www.uniprot.org/), combined, and used as database resulting in 29,558 protein sequences. Individual *.fasta entries per species are given (Table S3). The false discovery rates (FDR) were determined with the node Percolator [38] embedded in Proteome Discoverer (v 2.2) and we set the FDR threshold at a peptide level of 5%. The same threshold was set for the protein FDR (5%). Redundant proteins from the protein-coding database were automatically grouped in protein groups by applying the strict parsimony principle. Only the protein groups that explain at least one unique identified peptide were reported. Only the peptides that were not shared between different proteins or protein groups were used for the protein quantification through the Top3 approach implemented in the Proteome Discoverer (v 2.2). *GhoastKOALA* was used to assign KEGG orthology (KO) numbers of KEGG to the identified functions of identified protein sequences. A protein report from Proteome Discoverer with assigned taxa and functional information from KEEG are provided (Supplementary Material S4). Only pathways with sufficient coverage (>10%) on the total amount per sample were used for statistical analysis. For specific pathway abundances, only pathways with sufficient relative abundance (>0.01%) per sample were evaluated. Visualization and statistical analysis were carried out with the GraphPad Prism (v. 8.0.2) using unpaired multiple t-tests per row. A Pearson correlation was performed with in-house written R scripts (*Hmisc* package using the *rcorr* function).

### 2.6. Metabolomics

#### 2.6.1. Metabolite Extraction

For the analysis of short-chain fatty acids (SCFAs), the method of Han et al. was modified [33,39]. The sample was mixed with acetonitrile to a final concentration of 50% acetonitrile. SCFAs were derivatized with 0.5 volumes of 200 mM 3-nitrophenylhydrazine and 0.5 volumes of 120 mM N-(3-dimethylaminopropyl)-N′-ethylcarbodiimide hydrochloride in pyridine for 30 min at 40 °C. The mix was then diluted 1:50 in 10% acetonitrile.

#### 2.6.2. LC-MS/MS Measurement and Data Analysis 

An amount of 50 µL of the diluted SCFA derivatives was injected into the LC-MS/MS system. Chromatographic separation of SCFAs was performed on an Acquity UPLC BEH C18 column (1.7 µm, Waters, Eschborn, Germany) using H_2_O (0.01% formic acid, FA) and acetonitrile (0.01% FA) as the mobile phases. The column flow rate was set to 0.35 mL/min and the column temperature was set at 40 °C. The gradient elution was performed as follows: 2 min at 15% B, 15%–50% B in 15 min, and then held at 100% B for 1 min. Lastly, the column was equilibrated for 3 min at 15% B. Mass spectrometric analysis of metabolites was performed QTRAP^®^5500 (AB Sciex, Framingham, MA, USA). For identification and quantitation, a scheduled multiple reaction monitoring (MRM) method was used, with specific transitions for every SCFA. Peak areas were determined in Analyst^®^ Software (v. 1.6.2, AB Sciex) and areas for single SCFAs were exported. Normalization and statistics were performed with in-house written R scripts.

## 3. Results

### 3.1. Adaptation Phase of SIHUMIx under Continuous Cultivation Conditions

**Figure 2:**
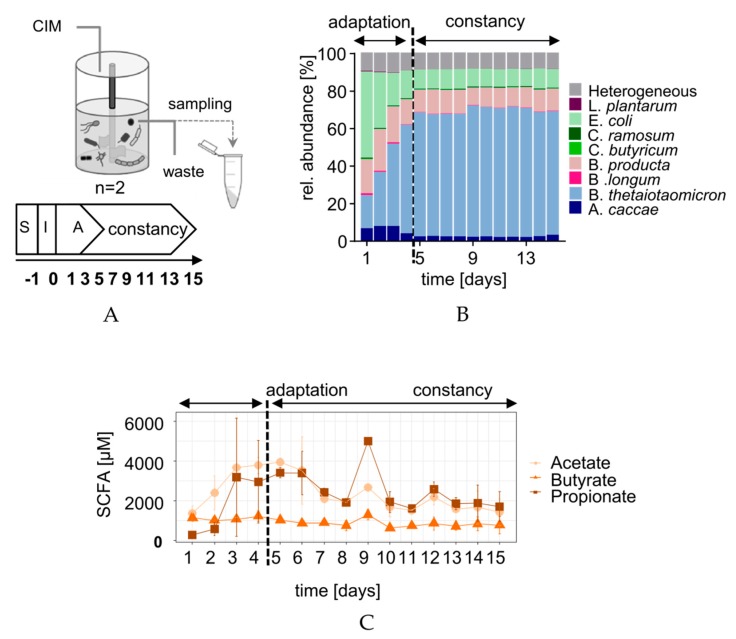
(**A**) Schematic overview of sample analysis to describe a stable functional state. *CIM* = Complex Intestinal Medium, S = sterile run, I = Inoculation, and A = adaptation. (**B**) The stacked bar graphs show taxa distribution over time (TT 24 h, *n* = 2) based on the relative protein abundance per species. (**C**) Absolute acetate, butyrate, and propionate concentrations over time.

Bioreactors (n = 6) were inoculated with 1 × 10^9^ cells/250 mL per species of the SIHUMIx bacteria and cultivated for five days (adaptation phase) until reaching a structural and functional constant state (Figure 2A) (Krause et al., in revision at the *Gut Microbes Journal*). To evaluate the species distribution of SIHUMIx, the community composition was analyzed based on the abundance of species-specific proteins [33,40]. In total (n = 90 samples), 7307 protein groups were identified. Protein group identification per sample is given (Supplementary Material Table S4). Within the first 24 h after inoculation, the relative abundance of *Clostridia* (*C. ramosum*, *C. butyricum*), *Lactobacillus* (*L. plantarum*), and *Bifidobacterium* (*B. longum*) clearly decreased, whereas the other SIHUMIx members, *Anerostipes* (*A. caccae*), *Bacteroides* (*B. thetaiotaomicron*), *Blautia* (*B. producta*) and *Escherichia* (*E. coli*), increased (Figure 2B). During the following four days, the abundance of *B. thetaiotaomicron* continued to increase, whereas the relative abundance of *A. caccae* and *E. coli* decreased. At the end of the adaptation phase (day 5), *B. thetaiotaomicron*, *B. producta*, *E. coli*, and *A. caccae* were the dominant members of SIHUMIx with 66%, 12%, 10.2%, and 2.7%, respectively.

To evaluate the metabolic activity of SIHUMIx, SCFA concentrations were measured daily (Figure 2C). The butyrate concentration was slightly higher on day one (1.1 mM) and stabilized on day five (1.02 mM). Acetate and propionate started at 1.3 mM and 0.3 mM, respectively, and increased until day five to 3.94 mM and 3.40 mM. At day 5, the concentration of acetate and propionate was 3.8 (SD = ±0.268) and 3.3 (SD = ±0.377) times higher when compared to butyrate. 

### 3.2. SIHUMIx Shows Slight Changes during Varying Transit Times

**Figure 3:**
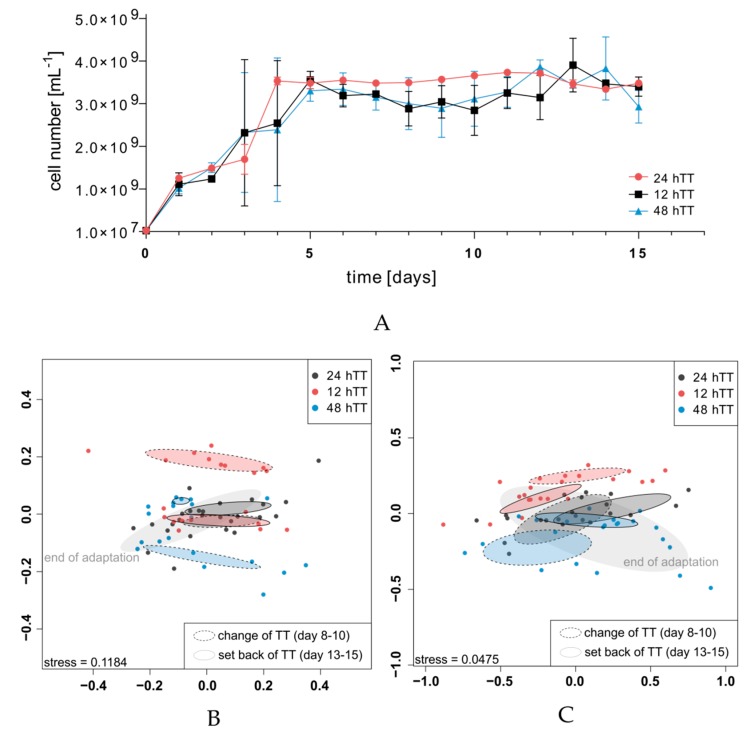
(**A**) Cell numbers of SIHUMIx. The NMDS plots show community dynamics during the experiment (Bray-Curtis dissimilarity) based on flow cytometry subpopulations (**B**) and absolute SCFA concentrations (**C**). The end of the adaptation of SIHUMIx is marked with a light grey ellipse. Control: dark grey ellipse with the dashed outline including days 8 to 10 and set back control: dark grey ellipse with the bold outline including days 13 to 15.

The adaptation phase was followed by the intermediary phase in whichTTs were shifted (day 6 to 10). In two bioreactors, the TT was set to 12 h and, in another set of two bioreactors, it was set to 48 h, respectively. On day 11, the TTs were set to 24 h again for all bioreactors and maintained until the end of the experiment on day 15 (Figure 1). The bacterial cell number was determined (Figure 3A). Compared to the end of the adaptation phase, the cell numbers stayed unchanged for 12 hTT, but decreased slightly at 24 hTT and 48 hTT.

The impacts on the community structure and SCFA production were followed with two fingerprinting methods: flow cytometric fingerprinting based on flow cytometric data and short-chain fatty acid analysis based on the SCFA concentrations. With dissimilarity analysis (visualized as non-metric multidimensional scaling (NMDS) plots), the effects of different TTs on the community structures were indicated. The end of the adaptation phase of all six bioreactors was included as a landmark for the following changes of both the community structures and the metabolic activities (Figure 3B,C, grey ellipse without outline, “end of adaptation”). Grouping refers to the different phases of the experiment (see legend in Figure 2B,C). All the Bray-Curtis Dissimilarity’s (BD) of flow cytometric data, which were used for the NMDS plotting, are given (Table S5). All groups were compared by pairwise ANOVA. R^2^ and *P* values of all group comparisons are given (Table S6).

Flow cytometric fingerprinting showed that SIHUMIx was affected by the increase and decrease of the TT regarding the community structure (Figure 3B). A slight difference was visible within the control, referring to days 8 to 10 of the control bioreactors, and the setback phase of the control, referring to days 13 to 15 of the control bioreactors. This indicates that there was a time-dependent structural shift of the community since TT did not change. The constancy spaces were determined for the two control bioreactors and a value of tr = 0.1447 (24 hTTI) was chosen as the accepted maximal intrinsic community variation (Figure S2). The proportion of the intrinsic variance between bioreactors and over time was small with regard to the result from pairwise ANOVA testing (control vs. set back control: R^2^ = 0.61) even though the changes were found to be insignificant (P adjusted = 0.515, Supplementary Material Figure S6). In contrast, a comparison of the communities revealed that, within the changes in the TT, the communities changed significantly (P adjusted = 0.004) during 12 hTT and 48 hTT, which indicates a TT-dependent shift of the community structure. The proportion of intrinsic variance between 12 hTT and 48 hTT (R^2^ = 6.36) was the highest calculated proportion of variance of all the calculated proportions of variances between the experimental groups. After the TT was set back to 24 h, the communities of the 12 hTT bioreactors reached a state comparable to the end of the adaptation. In contrast, after 48 hTT, the communities did not reach a state comparable to the end of the adaptation.

Additionally, short-chain fatty acid analysis revealed differences during the phases of varying TTs (Figure 3C). All Bray-Curtis Dissimilarity’s of SCFA concentrations, which were used for the NMDS plots, are given (Table S5). All the groups were compared by pairwise ANOVA testing. The *P* values of all the group comparisons are given (Table S6). During 12 hTT and 48 hTT, the SCFA composition was significantly changed (R^2^ = 7.33, P adjusted = 0.012), but reached a state comparable to the end of the adaptation afterward (end of adaptation vs. set back at 48 hTT R^2^ = 1.87, P adjusted = 0.191, end of adaptation vs. setback 12 hTT R^2^ = 1.93, P adjusted = 0.817). Only one further significant difference was found, namely between 12 hTT and the set back control (R^2^ = 5.23, P adjusted = 0.035), but the proportion of variance between bioreactors was smaller compared to 12 hTT vs. 48 hTT.

Pairwise ANOVA analysis revealed less significant differences between the groups based on the SCFA concentrations when compared to those based on flow cytometric data.

### 3.3. Stability Properties of SIHUMIx during Changed Retention Times

To analyze the stability properties of SIHUMIx during varying TTs, the constancy values for each bioreactor were quantified based on flow cytometric data [32]. Constancy describes the ability of bacterial communities to stay essentially unchanged. Therefore, an artificial community state was defined by calculating the mean of cell abundances per each gate of the 19 gates upon all samples from day five (end of the adaptation phase) to day 15 (n = 11) for both bioreactors with 24 hTT.

To calculate the community constancy space, a multidimensional dissimilarity space was considered, described by variations of community states and determined by Canberra Distance (CD). The maximum CD value was used to define the radius of the constancy space.

The constancy value ranges between zero and one, while a smaller value represents a smaller community variation, which indicates a relatively high constancy. We found that the two communities of the control bioreactors, which were run with 24 hTT in all phases, showed the smallest constancy values (control bioreactor 24 hTTI CD = 0.1447, control bioreactor 24 hTTII CD = 0.1235) after 15 days of cultivation. All constancy values are shown (Figure S2). The higher constancy value of the control bioreactors (CD = 0.1447, control bioreactor 24 hTTI) was used to define the threshold for accepted intrinsic community variation under continuous cultivation conditions. This threshold was set to investigate whether a structural deviation of SIHUMIx during a transition change increases to higher values, which would point to instability.

**Figure 4:**
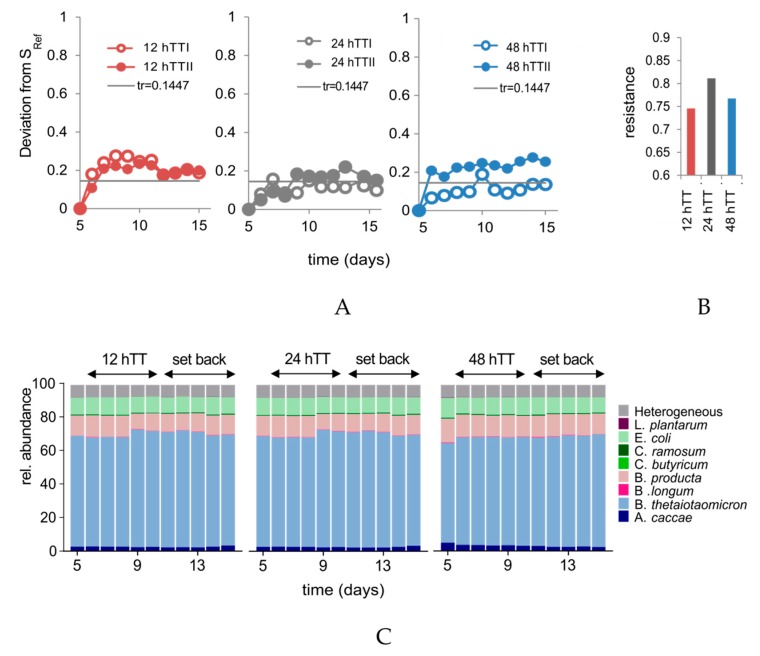
(**A**) The deviation of SIHUMIx from SRef. Tr = threshold of constancy space. Sref = reference state. (**B**) Resistance calculation based on flow cytometric data. (**C**) The stacked bar graphs show the taxa distribution of SIHUMx (12 h/24 h/48 h, *n* = 2) based on the abundance of species-specific proteins during the phase of changed TT and during the set back of TT afterward.

To describe the stability of the communities (i.e., resistance to TT changes), the deviation values of each bioreactor in the different experimental phases were compared to their reference states. As a reference state, the last state of the adaptation (day 5) was set per each bioreactor. Then the community deviation was followed (Figure 4A). If the deviation of the community is higher than the threshold value of the constancy space, the community does change structurally.

The deviation values of the control bioreactors were close to the threshold value after 15 days of cultivation, which indicates that the community deviation was similar to the intrinsic variation, calculated by the constancy space. For 12 hTT, the community deviated, with a maximum value of 0.275 (bioreactor 12 hTTI, at day 8) and 0.233 (bioreactor 12 hTTII, at day 10). The community states of the samples from bioreactor 48 hTTI showed at day 10, with a maximum deviation value of 0.188, the state that has a deviation higher than the threshold. In contrast, even with the same settings, bioreactor 48 hTTII deviated from its reference state immediately and reached the highest deviation value of 0.2765 at day 14.

Furthermore, resistance (RS) values for all the bioreactors were calculated based on the deviation values. The RS values of all the bioreactors were within the range of 0.72 to 0.85, which indicates a relatively high resistance against changing TTs (all the resistance values are shown in the Supplementary Material, Figure S2). The mean RS values for the duplicate bioreactors with the same TTs are shown in Figure 4B. The control showed the highest RS (RS = 0.8114), which is followed by the 48 hTT (RS = 0.7674) and the 12 hTT (RS = 0.7459) bioreactors.

### 3.4. Changes in TT are Associated with Differences in Specific Pathways

To investigate whether the structural differences were associated with changes in species distribution or function, the metaproteomic analysis was performed. There were no changes in the taxa distribution of SIHUMIx in all the bioreactors since the end of the adaptation (day 5), despite the varied TTs (Figure 4C). Relative species abundances are given (Table S7).

**Figure 5:**
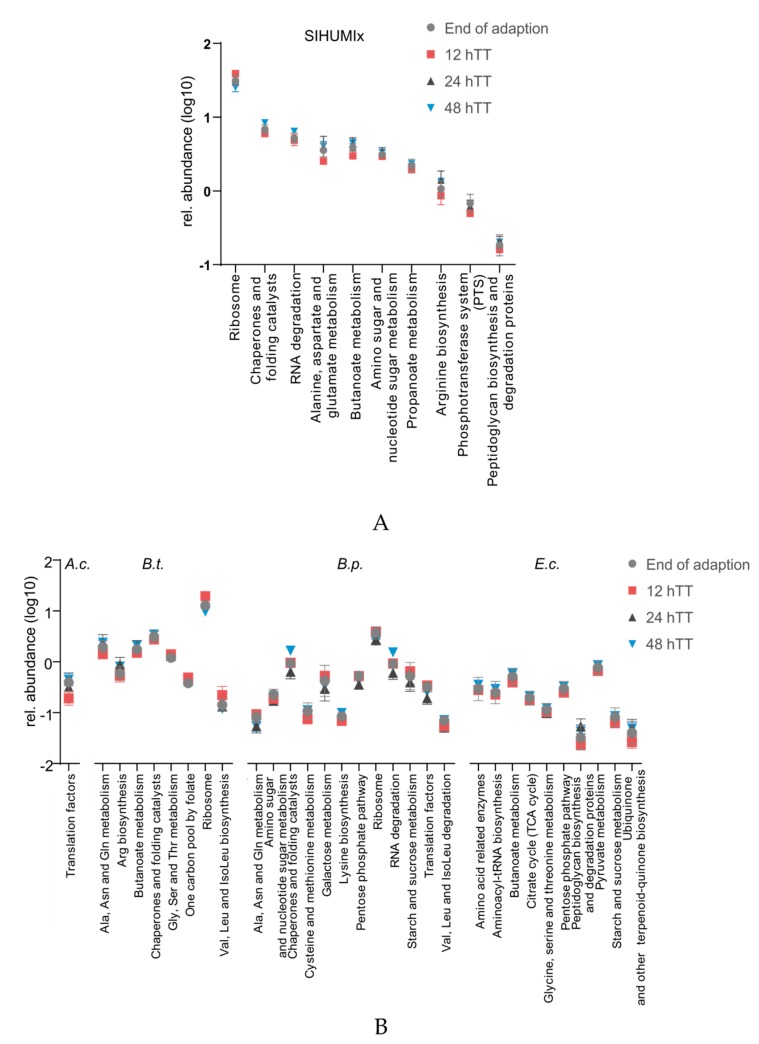
(**A**) Log transformed relative abundance of all proteins identified per pathway for SIHUMIx. (**B**) Relative pathway abundances of *A. caccae, B. thetaiotaomicron*, *B. producta*, and *E. coli* based on the average of six for the end of adaptation (day 5) or four for 12 h/24 h/48 h (day 9 and 10).

Although relative species abundance was not affected (Figure 4C), differences were found in specific functions of SIHUMIx (Figure 5A). Differences were observed between 12 hTT and 48 hTT for the following KEGG pathways: ribosome (*p* = 0.0013), chaperones and folding catalysts (*p* = 0.0008), RNA degradation (*p* = 0.0311), alanine, aspartate, and glutamate metabolism (*p* = 0.002), butanoate metabolism (*p* = 0.0027), amino sugar and nucleotide sugar metabolism (*p* = 0.036), propanoate metabolism (*p* = 0.049), arginine biosynthesis (*p* = 0.0111), phosphotransferase system (PTS) (*p* = 0.004), and peptidoglycan biosynthesis and degradation proteins (*p* = 0.0016). All the pathways were less abundant at 12 hTT compared to 48 hTT, except for the ribosome, which were more abundant at 12 hTT. On a species level, slight changes in relative abundances were found for 27 pathways identified in *A. caccae*, *B. thetaiotaomicron*, *B. producta*, and *E. coli,* which are the most abundant species in the SIHUMIx community (Figure 5B).

### 3.5. Changes in TT are Associated with Differences in SCFA Concentrations

**Figure 6:**
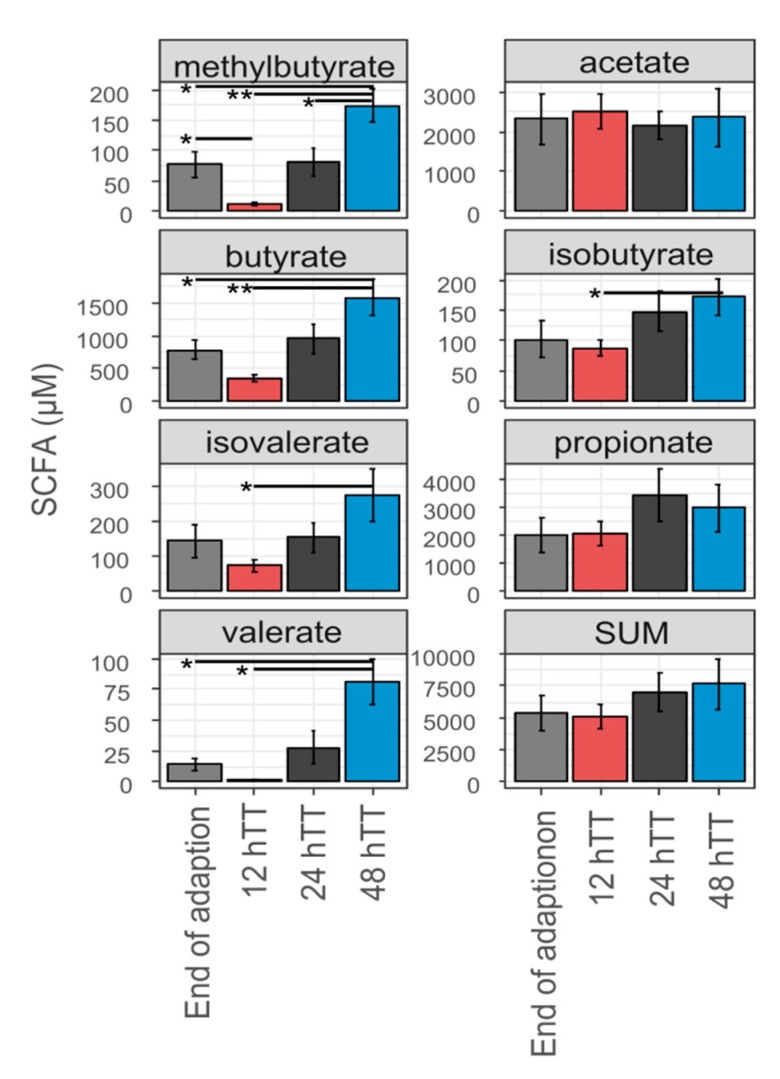
SCFA and branched-chain fatty acids (BCFA) concentrations under different transit times based on the average of six for the end of adaptation (day 5) or four for 12 h/24 h/48 h (day 9 and 10). *p* value was calculated with multiple *t*-test (using no *p*-value adjustment) between 12 hTT and 48hTT. * *p* < 0.05, ** *p* < 0.001.

In addition to differences in specific functions, SCFA concentrations changed during the varying TTs (Figure 6).

The absolute concentrations of butyrate (*p* = 0.0049) and valerate (*p* = 0.0404) were lower at 12 hTT compared to 48 hTT. This was also true for the BCFA methylbutyrate (*p* = 0.0012), isobutyrate (*p* = 0.039), and isovalerate (*p* = 0.0381), which decreased at 12 hTT compared to 48 hTT. The amount of total SCFA and the concentration of acetate and propionate were not affected by changes in TT.

## 4. Discussion

The colon transit time (TT) has been described as a driving force that affects the composition and metabolism of complex intestinal communities, but the results differ between studies [15,16,20,21]. We recently established an extended simplified human intestinal microbiota (SIHUMIx) for bioreactor used to investigate physiological stressors on a species-specific level (Krause et al., in revision at the *Gut Microbes Journal*). Since the effects of varying TT on structure and function are not fully understood, we investigated the effects of three physiological colonic TT (48 h, 24 h, 12 h) on SIHUMIx. SIHUMIx was grown for 15 days in a colon simulating bioreactor system. Daily samples were analyzed with metaproteomics, SCFA analysis, and two fingerprinting techniques using flow cytometry data and SCFA concentrations to visualize the effect of changing TT on the community structure and function of SIHUMIx. This study shows that the structure of SIHUMIx was only slightly affected by changing TT and showed high stabilizing abilities after adapting to the bioreactor conditions. Functional changes during changing TT are only partly in accordance with changes found for complex communities, i.e., it could be associated with the relative abundance changes of *B. thetaiotaomicron*, *B. producta*, and *E. coli*, which are the most abundant members of SIHUMIx.

### 4.1. SIHUMIx Shows Slight Changes During Varying Transit Times

TT in the bioreactor system can be influenced by differences in pump speed and clogging of the tubes. In order to prevent this (i), all medium pumps were calibrated before the experiments to assure no technical differences between the individual feed pumps and (ii) medium bottles were constantly stirred during the experiment to prevent particle formation.

The total cell number of SIHUMIx fluctuated during the whole experiment, but stayed mainly unchanged at 12 hTT, whereas the total cell numbers at the 24 hTT (control) and 48 hTT bioreactors slightly decreased during varying TTs (Figure 3A). The effect of the total cell numbers during 12 hTT is in contrast with studies conducted on complex bacterial communities [15]. In this study, the total cell numbers were not affected differently by varying TTs.

The different TTs also showed changes in the cytometric community structure using flow cytometric fingerprinting. During the phase of changed TT, communities of 48 hTT and 12 hTT were clearly different from each other but not to the control. To determine whether the structural differences in the SIHUMIx communities were higher than the normal community variation in the control at 24 hTT, the deviation of community states that the end of the adaptation was calculated. Accelerating the TT affected the community structure at 12 hTT. Both bioreactor communities deviated out of the constancy space. For the longer 48 hTT, the duplicate communities behaved differently. One of the 48 hTT bioreactors deviated from the reference state and out of the constancy space, whereas the other mostly stayed unchanged. Since only one replicate bioreactor deviated from the reference state, we assume that the microbial community in this bioreactor was not constant at day five. Although we expected that SIHUMIx reaches a constant state on day five (Krause et al., in revision at the *Gut Microbes Journal*), the adaptation phase should be prolonged to seven days in order to assure balanced growth in future studies.

In addition, with regard to larger structural deviations (Figure 4A) and significant community variations (Figure 3B,C), smaller resistance values were found in the groups of 12 hTT and 48 hTT but they were close to the values of controls. This shows that SIHUMIx is highly resistant against variation of TTs. During faster TTs, cell growth is likely to be supported since absolute cell numbers were also the highest at 12 hTT.

Bacterial cells modify their metabolism during the cell cycle and, thus, the community structure changes. Since flow cytometric fingerprinting is based on the measurement of cell size and DNA content, structural changes caused by different TTs can also be related to changes in the division states of community members or to a shift in species abundances. Metaproteomics revealed that species abundances stayed unchanged, which indicates that the communities were clearly not affected based on phylogenetic affiliation but by cell cycle activities. All the species, including the slow-growing bacterium *L. plantarum*, remained in the bioreactor system until the end of the experiment on day 15 [41].

### 4.2. Changes are Associated with Cell Division

In bacterial cell division, the final step is dependent on certain cell size. Bacterial cells need to at least double their biomass before division in order to prevent biomass losses. Individual cell sizes vary within a population and exhibit intrinsic variability, even under constant growth conditions [42]. Medium TT directly affects the availability of the medium ingredients. Carbon availability is higher at a shorter TT and, therefore, is likely to cause a functional change and faster growth. This functional change might be associated with changes in cell size and, hence, the time of division of cell subpopulations.

Functional differences were found for specific pathways in the KEGG sub-roles: translation, folding, sorting and degradation, membrane transport, and amino acid and glycan metabolism, which are relevant for DNA replication and cell division (Figure 5A). During cell division, bacterial cells need to replicate two main components: the chromosomes and the peptidoglycan cell wall [43]. In fact, the glycan metabolism of SIHUMIx was affected differently during varying TTs. During the 12 hTT, the relative protein abundance of the pathways peptidoglycan biosynthesis and degradation proteins decreased. It is probable that, at 12 hTT, more alternative carbon and nitrogen sources were available and cell wall synthesis could be maintained without energy consumption recycling of peptidoglycans [44], but it is still unknown exactly how peptidoglycan chains are synthesized [43]. When bacteria are shifted from low to high nutrient availability, the expression of ribosomal RNA and ribosomal proteins is likely to accelerate [45]. Hence, in this study, the relative abundance of the proteins related to the KEGG pathway ribosome of SIHUMIx was higher at 12 hTT compared to 48 hTT. Apparently, this shift was slightly higher for *B. thetaiotaomicron* than *B. producta.* For other members of SIHUMIx, changes in relative abundances of ribosomal proteins were not assigned.

### 4.3. A Faster Transit Time Slightly Reduces Butyrate Metabolism of SIHUMIx And Favors Protein Fermentation

Short-chain fatty acid analysis revealed changes between 12 hTT and 48 hTT, even though changes on the functional level were smaller than those on the structural level. These changes were caused by a decrease in butyrate, methylbutyrate, isovalerate, and valerate concentrations at 12 hTT, whereas total SCFA concentrations were not significantly changed (Figure 6). In accordance with the SCFA analysis, the butyrate metabolism also decreased, which was shown by less protein abundance of butanoate metabolism at 12 hTT (Figure 5A). The reduction of butanoate metabolic proteins was specifically shown for *B. thetaiotaomicron* and *E. coli* (Figure 5B).

Furthermore, we found a decrease in the amino acid metabolism of SIHUMIx at 12 hTT by the less relative abundance of alanine, aspartate, and glutamate metabolism and arginine biosynthesis. Together with a reduced butanoate metabolism, this indicates reduced carbohydrate fermentation at faster TTs. In contrast to carbohydrate fermentation, the fermentation of proteins and amino acids resulted in the production of BCFA. The concentrations of the BCFA methylbutyrate, isovalerate, and isobutyrate increased (Figure 6), which indicates that protein fermentation was favored at faster TT. These findings are in accordance with previous results and strengthen the hypothesis that protein fermentation is favored with faster TT [46,47]. Functional changes in the pathway abundances of low abundant SIHUMIx bacteria were not detected. This is most likely due to the low proteome coverage of low abundant species (less than 1%), which is still a bottleneck in metaproteome analysis [48,49].

**Figure 7:**
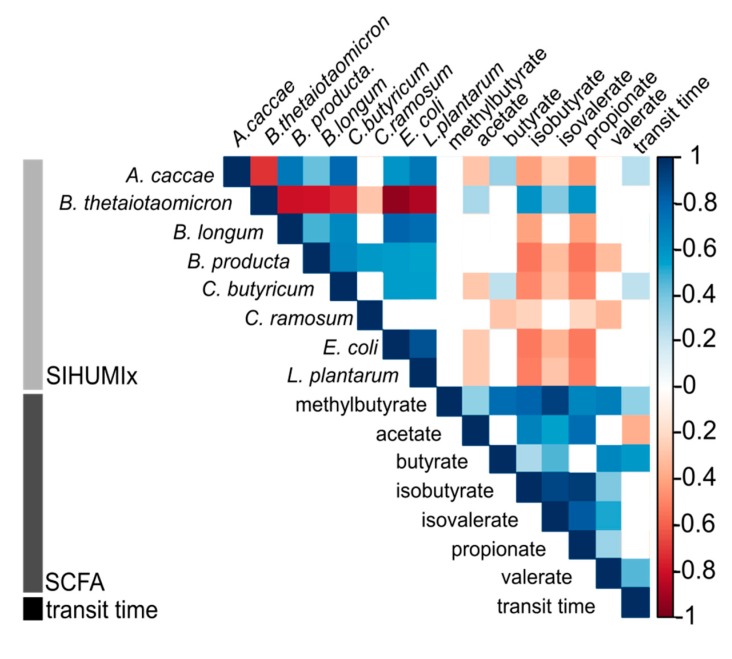
Correlation between relative species abundance, SCFA, and transit time.

Fermentation end products are well studied, but the association between a specific substrate and SCFA formation is not well understood since several amino acids can be used for both SCFA and BCFA synthesis [50]. Although the absolute acetate concentration did not significantly increase at 12 hTT, correlation analysis revealed that acetate was slightly negatively correlated with the TT (Figure 7). The final steps of butyrate synthesis can be realized by two different enzymes: butyrate kinase, which uses butyryl-CoA, or butyryl-CoA:acetate-CoA transferase, which uses acetate to form butyrate. The latter has been suggested as the preferred route used by intestinal bacteria [51]. It has been shown that these enzymes are possessed simultaneously or depend on external metabolites [51,52]. It is likely that the decreased butyrate metabolism caused an increased acetate availability at 12 hTT.

### 4.4. B. thetaiotaomicron is Associated with SCFA and BCFA Production

Saccharolytic activity in the human intestine mainly concerns the breakdown of carbohydrates via the Embden-Meyerhof-Parnas pathway. At first, glucose is metabolized to pyruvate and further into SCFA. Genera that produce SCFA are known to be primarily *Bacterioides spp*., *Lactobacillus spp.*, and *Bifidobacterium spp*. [8]. The correlation analysis of the SIHUMIx species abundance with the absolute concentrations of fermented SCFA products revealed that propionate could be strongly associated with *B. thetaiotaomicron* but not with *L. plantarum* or *B. longum* (Figure 7).

*B. thetaiotaomicron* was also positively correlated with isobutyrate and isovalerate. Isobutyrate and isovalerate can result from valine and leucine degradation. Both amino acids are utilized in the intestinal microbiota [53] Previous results correlated the production of BCFA only with low abundant species in a complex microbial community since they were found to be mainly responsible for protein degradation [21]. In our study, the production of BCFAs was correlated with the most abundant member of SIHUMIx *B. thetaiotaomicron*. However, only the production of propionate has been described to date [46]. At approximately 65%, *B. thetaiotaomicron* is the most abundant member of SIHUMIx. Mucus-associated bacterium *B. thetaiotaomicron* is the only member of SIHUMIx, with the ability to degrade mucin. Mucin is a glycoprotein, which can serve as a source for both carbohydrate and protein fermentation. This might provide an ecological advantage in nutrient accessibility and account for its high abundance [54,55]. Although mucin availability varied during the different TTs, the abundance of *B. thetaiotaomicron* was not affected.

## 5. Conclusions

Even though simplified bacterial communities are expected to be less stable against environmental stressors, SIHUMIx showed remarkable structural and functional resistance against varying physiological TTs. Moreover, correlation analysis revealed little correlation between species abundances or SCFA concentration and the TT, which indicates a high resistance of SIHUMIx against varying physiological TTs. Fingerprinting tools provided a fast and reproducible approach to assess microbial community dynamics and revealed slight changes in the community structure of SIHUMIx during varying TTs. According to our metaproteomics data, these changes were associated with differences in membrane transport, glycan and protein metabolism, and, consequently, absolute SCFA and BCFA concentrations. *B. thetaiotaomicron* was identified to be affected in terms of butyrate metabolism, which has an important implication concerning intestinal health. Despite the limitation due to the relatively low abundant species, this study shows that the low complexity of SIHUMIx allowed the description of the effect of physiological stressors on a species-specific level.

## Supplementary Materials

The following are available online at https://www.mdpi.com/2076-2607/7/12/641/s1, Figure S1: Master cell gate template for flow cytometric fingerprinting, Figure S2: Constancy space and resistance values per bioreactors based on flow cytometric data, Table S1: Additional information about SIHUMIx, Table S2: Growth media (Brain-Heart-Infusion, BHI), Complex intestinal medium, CIM), and growth conditions, Table S3: Protein-coding sequences per species, Table S4: Protein report of metaproteomic analysis, Table S5: Bray-Curtis Dissimilarity’s of NMDS evaluation of flow cytometric data and SCFA concentrations, Table S6: P values from Pairwise ANOVA of flow cytometric data and SCFA concentrations, Table S7: Relative species abundance of SIHUMIx.
