# Supplementary material for: The Simplified Human Intestinal Microbiota (SIHUMIx) Shows High Structural and Functional Resistance against Changing Transit Times in In Vitro Bioreactors
Source: Microorganisms. 2019 Dec 3;7(12):641. doi: 10.3390/microorganisms7120641 (PMC6956075; doi:10.3390/microorganisms7120641)
Supplement: Supplementary file 1 [file microorganisms-07-00641-s001.zip › Supplementary_material_F1_master_cell_gate.docx]

**Supplementary Material Figure S1: Master cell gate template for flow cytometric fingerprinting of SIHUMIx.**


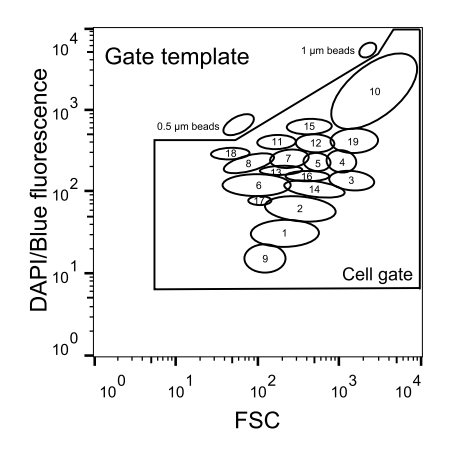


Figure S1: Cells were measured after fixation and DAPI staining. The gate template combines 19 gates to measure cell number variances per subcommunity.
