# Supplementary material for: The Simplified Human Intestinal Microbiota (SIHUMIx) Shows High Structural and Functional Resistance against Changing Transit Times in In Vitro Bioreactors
Source: Microorganisms. 2019 Dec 3;7(12):641. doi: 10.3390/microorganisms7120641 (PMC6956075; doi:10.3390/microorganisms7120641)
Supplement: Supplementary file 1 [file microorganisms-07-00641-s001.zip › Supplementary_material_F2_Constancy_resistance_values.pdf]

Supplementary Material Figure S2: Constancy and resistance values of each bioreactor

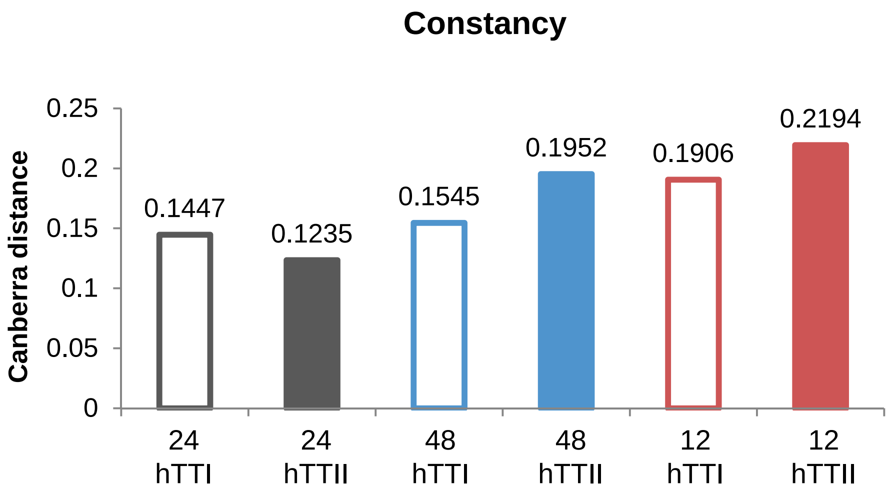

Figure S2.1. Constancy per each bioreactor based on Canberra distance. Cytometric data from day 5 to day 15 are used to define the constancy space per reactor, while the radius (threshold) of the constancy space is calculated to indicate the constancy value (tr).

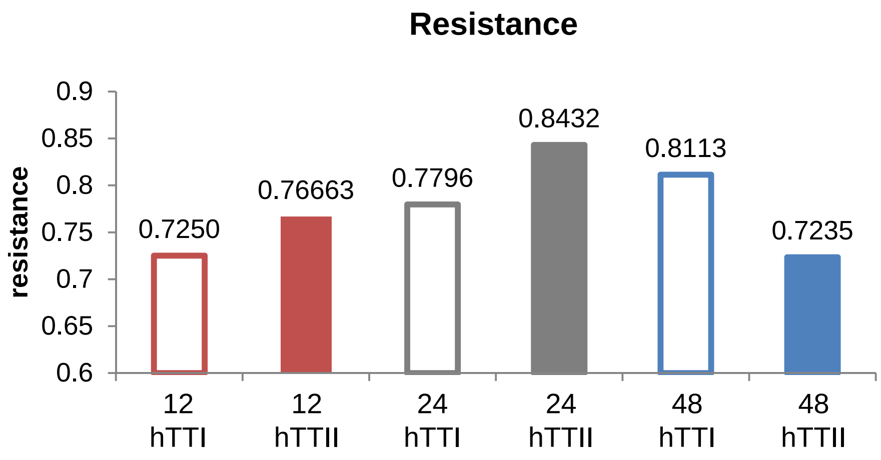

Figure S2.2. Resistance values per each reactor. The community state at the end of the adaptation (day 5) was compared with the community states of the following days. The deviation values (Canberra distance) were used to calculate the resistance.
