# Supplementary material for: The Simplified Human Intestinal Microbiota (SIHUMIx) Shows High Structural and Functional Resistance against Changing Transit Times in In Vitro Bioreactors
Source: Microorganisms. 2019 Dec 3;7(12):641. doi: 10.3390/microorganisms7120641 (PMC6956075; doi:10.3390/microorganisms7120641)
Supplement: Supplementary file 1 [file microorganisms-07-00641-s001.zip › Supplementary_material_S1_SIHUMIx.docx]

**Supplementary Material Table S1: Additional Information about SIHUMIx**

We recently established SIHUMIx as *in vitro* model for bioreactor use for the first time (Krause et al., in submission). We could show that the consortium in that composition reproducibly established in the bioreactor system until it reached the ability to stay essentially unchanged. This is an important criterion for the investigation of environmental stressors in an *in vitro* model, since studies on complex bacterial communities have been reported to lack true replication [1,2]. Functions that are known to be fulfilled by each species are given in Table S1.

Table S1.1: Overview of important functions of SIHUMIx species

| **species** | **phylum** | **secondary**  **bile acids conversion** | **function in the human gut** |
| --- | --- | --- | --- |
| *Anaerostipes caccae*  *(=Eubacterium entericum)* | Firmicutes | yes | short chain fatty acids producer[3] |
| *Bacteroides thetaiotaomicron* | Bacteroidetes | yes | mucosal barrier reinforcement  immune system modulation  nutrients metabolism[4] |
| *Bifidobacterium longum* | Actinobacteria | yes | produces acetate  catabolism of oligosaccharides[5] |
| *Blautia producta* | Firmicutes | yes | glucose fermentation  among the most abundant members[6,7][5] |
| *Clostridium butyricum* | Firmicutes | no | short chain fatty acids producer  dehydroxylation of bile acids[8,9] |
| *Clostridium ramosum* | Firmicutes | no | conversion of bilirubin to urobilinogen  dehydroxylation of bile acids [10] |
| *Escherichia coli* | Proteobacteria | no | metabolism of high spectrum of glycoconjugates[11] |
| *Lactobacillus plantarum* | Firmicutes | yes | immunomodulation  enhancement of the epithelial barrier functions[12] |

1. Liu, Z.; Cichocki, N.; Bonk, F.; Gunther, S.; Schattenberg, F.; Harms, H.; Centler, F.; Muller, S. Ecological stability properties of microbial communities assessed by flow cytometry. *mSphere* **2018**, *3*.

2. Fernandez, A.; Huang, S.; Seston, S.; Xing, J.; Hickey, R.; Criddle, C.; Tiedje, J. How stable is stable? Function versus community composition. *Appl Environ Microbiol* **1999**, *65*, 3697-3704.

3. Schwiertz, A.; Hold, G.L.; Duncan, S.H.; Gruhl, B.; Collins, M.D.; Lawson, P.A.; Flint, H.J.; Blaut, M. Anaerostipes caccae gen. Nov., sp. Nov., a new saccharolytic, acetate-utilising, butyrate-producing bacterium from human faeces. *Syst Appl Microbiol* **2002**, *25*, 46-51.

4. Zocco, M.A.; Ainora, M.E.; Gasbarrini, G.; Gasbarrini, A. Bacteroides thetaiotaomicron in the gut: Molecular aspects of their interaction. *Dig Liver Dis* **2007**, *39*, 707-712.

5. Schell, M.A.; Karmirantzou, M.; Snel, B.; Vilanova, D.; Berger, B.; Pessi, G.; Zwahlen, M.C.; Desiere, F.; Bork, P.; Delley, M.*, et al.* The genome sequence of bifidobacterium longum reflects its adaptation to the human gastrointestinal tract. *Proc. Natl. Acad. Sci. U. S. A.* **2002**, *99*, 14422-14427.

6. Rajilic-Stojanovic, M.; de Vos, W.M. The first 1000 cultured species of the human gastrointestinal microbiota. *FEMS Microbiol Rev* **2014**, *38*, 996-1047.

7. Liu, C.; Finegold, S.M.; Song, Y.; Lawson, P.A. Reclassification of clostridium coccoides, ruminococcus hansenii, ruminococcus hydrogenotrophicus, ruminococcus luti, ruminococcus productus and ruminococcus schinkii as blautia coccoides gen. Nov., comb. Nov., blautia hansenii comb. Nov., blautia hydrogenotrophica comb. Nov., blautia luti comb. Nov., blautia producta comb. Nov., blautia schinkii comb. Nov. And description of blautia wexlerae sp. Nov., isolated from human faeces. *Int J Syst Evol Microbiol* **2008**, *58*, 1896-1902.

8. Midtvedt, T.; Gustafsson, B.E. Microbial conversion of bilirubin to urobilins in vitro and in vivo. *Acta Pathol Microbiol Scand B* **1981**, *89*, 57-60.

9. Mo, S.; Kim, B.S.; Yun, S.J.; Lee, J.J.; Yoon, S.H.; Oh, C.H. Genome sequencing of clostridium butyricum dku-01, isolated from infant feces. *Gut Pathog* **2015**, *7*, 8.

10. Mackie, R.I.; Sghir, A.; Gaskins, H.R. Developmental microbial ecology of the neonatal gastrointestinal tract. *Am J Clin Nutr* **1999**, *69*, 1035S-1045S.

11. Alteri, C.J.; Mobley, H.L. Escherichia coli physiology and metabolism dictates adaptation to diverse host microenvironments. *Curr Opin Microbiol* **2012**, *15*, 3-9.

12. Meijerink, M.; van Hemert, S.; Taverne, N.; Wels, M.; de Vos, P.; Bron, P.A.; Savelkoul, H.F.; van Bilsen, J.; Kleerebezem, M.; Wells, J.M. Identification of genetic loci in lactobacillus plantarum that modulate the immune response of dendritic cells using comparative genome hybridization. *PLoS One* **2010**, *5*, e10632.
