# Supplementary material for: The Simplified Human Intestinal Microbiota (SIHUMIx) Shows High Structural and Functional Resistance against Changing Transit Times in In Vitro Bioreactors
Source: Microorganisms. 2019 Dec 3;7(12):641. doi: 10.3390/microorganisms7120641 (PMC6956075; doi:10.3390/microorganisms7120641)
Supplement: Supplementary file 1 [file microorganisms-07-00641-s001.zip › Supplementary_material_S2_growth_conditions.docx]

**Supplementary Material Table S2: Growth media (Brain-Heart-Infusion; BHI), Complex intestinal medium; CIM) and growth conditions**

Cultivation of SIHUMIx as single strains was performed in Brain-Heart-Infusion (BHI) medium under anaerobic conditions. All ingredients are given in Table S1.1.

Table S2.1: supplemented Brain-Heart-Infusion medium (BHI) storage at 4 C

| **Ingredient** | **Quantity [g or mL/L]** | **supplier** |
| --- | --- | --- |
| Brain-Heart-Infusion | 37 | Roth |
| L-cysteine hydrochloride | 0.5 | Biochemica |
| Resazurin | 0.001 | MP biomedicals |
| Vitamin K hemin solution | 10 | Becton Dickinson |
| Yeast extract | 5 | Chemsolute |

BHI medium was aliquoted into Hungate tubes and closed with a butyl cap. Before autoclaving Hungates were purged with nitrogen and stored at 4 C until use. Hungates were inoculated 1:10 with freshly thawed bacterial cells and incubated at 37°C and 175 rpm.

For the experiment SIHUMIx was continuously cultivated in a Multifors 2 bioreactor system (Infors, Switzerland) with six parallel 250 mL culture bioreactors filled with complex intestinal medium (CIM). CIM was slightly modified after McDonald et al. (2013) and all ingredients are given in Table S1.2 [1].

Table S2.2: Complex intestinal medium (CIM): Medium formulation was adopted from McDonald *et al.* (2013). PH was adjusted to pH 6.7 with NaOH, storage at 4 C

| **Ingredient** | **Quantity [g/L]** | **supplier** |
| --- | --- | --- |
| Arabinogalactan (larch wood) | 2 | Sigma-Aldrich |
| Bile Acids sodium salt | 0.5 | Sigma-Aldrich |
| Calcium chloride x 2 H_2_O | 0.01 | Merck |
| Casein peptone (pancreatic) | 4.3 | Roth |
| Di-Potassium hydrogen phosphate | 0.04 | Roth |
| Hemin (bovine) | 0.005 | Sigma-Aldrich |
| Inulin | 1 | Serva |
| L-cysteine hydrochloride | 0.5 | Biochemica |
| Magnesium sulfate | 0.01 | Roth |
| Menadione | 0.001 | Sigma-Aldrich |
| Mucin (porcine gastric Type II) | 4 | Sigma-Aldrich |
| Pectin, citrus peel | 2 | Sigma-Aldrich |
| Potassium di-hydrogen phosphate | 0.04 | Roth |
| Sodium chloride | 0.72 | Roth |
| Sodium hydrogen carbonate | 2 | Roth |
| Starch, wheat | 5 | Roth |
| Xylo-oligosaccharide (corn) | 2 | Roth |
| Yeast extract | 2 | Chemsolut |

For sterile autoclaving the bioreactors were filled with VE-water as the CIM could not be autoclaved. After autoclaving the water was removed and the bioreactors were filled with 250 mL sterile CIM, respectively, by pumping. Settling of bacterial cells was prevented by constant stirring at 150 rpm. The bioreactor system was maintained under anaerobic conditions by continuously gassing the bioreactors as well as the reservoir bottles (medium and NaOH) with sterile nitrogen. To make sure the system was sterile after the setting up the system was run under experimental conditions (37°C, 150 rpm, no medium feed) for 24 h as a sterility control. For this purpose fermentation the antifoam probe was used to constantly remove excess medium keeping the bioreactor volume at 250 mL.

1. McDonald, J.A.; Schroeter, K.; Fuentes, S.; Heikamp-Dejong, I.; Khursigara, C.M.; de Vos, W.M.; Allen-Vercoe, E. Evaluation of microbial community reproducibility, stability and composition in a human distal gut chemostat model. *J Microbiol Methods* **2013**, *95*, 167-174.
