# Supplementary material for: The Simplified Human Intestinal Microbiota (SIHUMIx) Shows High Structural and Functional Resistance against Changing Transit Times in In Vitro Bioreactors
Source: Microorganisms. 2019 Dec 3;7(12):641. doi: 10.3390/microorganisms7120641 (PMC6956075; doi:10.3390/microorganisms7120641)
Supplement: Supplementary file 1 [file microorganisms-07-00641-s001.zip › Supplementary_material_S3_Protein_coding_sequences.docx]

**Supplementary Material Table S3: Protein coding sequences of SIHUMIx used for Metaproteomics**

The protein coding sequences were downloaded from the UniProt (<http://www.uniprot.org/>) server of the eight SIHUMIx strains separately, combined and used as database resulting in 29,558 protein coding sequences. Individual .fasta entries per species are given in Table S3.

Table S3: Individual .fasta entries per species

| **Species** | **fasta_entries** |
| --- | --- |
| *Bacteroides thetaiotaomicron* | 4782 |
| *Escherichia coli* | 4307 |
| *Anaerostipes caccae* | 3743 |
| *Erysipelatoclostridium ramosum* | 3166 |
| *Blautia sp.* | 4502 |
| *Lactobacillus plantarum* | 3088 |
| *Clostridium butyricum* | 4245 |
| *Bifidobacterium longum* | 1725 |
