# Supplementary material for: The Simplified Human Intestinal Microbiota (SIHUMIx) Shows High Structural and Functional Resistance against Changing Transit Times in In Vitro Bioreactors
Source: Microorganisms. 2019 Dec 3;7(12):641. doi: 10.3390/microorganisms7120641 (PMC6956075; doi:10.3390/microorganisms7120641)
Supplement: Supplementary file 1 [file microorganisms-07-00641-s001.zip › Supplementary_material_S6_p_values_from_Pairwise_Anova_of_BD_values.docx]

Supplementary Material Table S6: Evaluation of differences of SIHUMIx composition and metabolic activity during varying transit times. Grouping refers to the phases of cultivation (Figure 2). Samples of every phase of the experiment were compared to every phase (duplicates of each transit time/phase) based on relative cell abundances per subcommunity assessed by flow cytometry and SCFA concentrations (pairwise ANOVA) (Fig. 2B,2C).

|  | **Flow cytometry data** | | **SCFA concentrations** | |
| --- | --- | --- | --- | --- |
| **group** | **R^2^** | **FDR-adj.**  **P-value** | **R^2^** | **FDR-adj.**  **P-value** |
| end of adaptation vs x | 1.40360698 | 0.241 | 0.62042306 | 0.52 |
| end of adaptation vs control | 1.0660198 | 0.355 | 0.80487831 | 0.45 |
| end of adaptation vs set back control | 1.84089167 | 0.158 | 0.37991981 | 0.681 |
| end of adaptation vs 48 hTT | 2.1269339 | 0.132 | 1.91196049 | 0.162 |
| end of adaptation vs set back 48 hTT | 2.65974642 | 0.031 | 1.86569632 | 0.191 |
| end of adaptation vs 12 hTT | 3.30101086 | 0.038 | 1.68412942 | 0.195 |
| end of adaptation vs set back 12 hTT | 1.3195252 | 0.231 | 0.19373408 | 0.817 |
| x vs control | 0.58850513 | 0.645 | 0.64650263 | 0.528 |
| x vs set back control | 1.0553506 | 0.362 | 0.75330396 | 0.478 |
| x vs 48 hTT | 2.8480265 | 0.051 | 2.25840232 | 0.103 |
| x vs set back 48 hTT | 2.14336871 | 0.091 | 2.51690922 | 0.081 |
| x vs 12 hTT | 3.77486378 | 0.016 | 4.08897632 | 0.031 |
| x vs set back 12 hTT | 1.30023698 | 0.268 | 0.22501841 | 0.855 |
| control vs set back control | 0.5149665 | 0.617 | 1.75878553 | 0.21 |
| control vs 48 hTT | 1.85102061 | 0.159 | 2.08286324 | 0.145 |
| control vs set back 48 hTT | 4.59286377 | 0.049 | 0.96874488 | 0.318 |
| control vs 12 hTT | 3.26799721 | 0.028 | 1.83156161 | 0.171 |
| control vs set back 12 hTT | 0.75859839 | 0.458 | 0.83636524 | 0.381 |
| set back control vs 48 hTT | 3.15407117 | 0.049 | 1.79655186 | 0.174 |
| set back control vs set back 48 hTT | 6.07864468 | 0.002 | 4.167235 | 0.071 |
| set back control vs 12 hTT | 2.52293958 | 0.078 | 5.27327154 | 0.035 |
| set back control vs set back 12 hTT | 0.56469028 | 0.578 | 0.75064528 | 0.403 |
| 48 hTT vs set back 48 hTT | 4.86428562 | 0.006 | 2.51586905 | 0.136 |
| 48 hTT vs 12 hTT | 6.356198 | 0.004 | 7.32964267 | 0.012 |
| 48 hTT vs set back 12 hTT | 2.62101447 | 0.097 | 2.85577734 | 0.091 |
| set back 48 hTT vs 12 hTT | 4.43108129 | 0.008 | 3.07396805 | 0.078 |
| set back 48 hTT vs set back 12 hTT | 5.74617429 | 0.006 | 3.30310582 | 0.086 |
| 12 hTT vs set back 12 hTT | 3.35790139 | 0.036 | 3.55410971 | 0.084 |

^1^x= refers to the the early days per phase (all bioreactors day 6, 7,11 and 12)
