# Supplementary material for: The Simplified Human Intestinal Microbiota (SIHUMIx) Shows High Structural and Functional Resistance against Changing Transit Times in In Vitro Bioreactors
Source: Microorganisms. 2019 Dec 3;7(12):641. doi: 10.3390/microorganisms7120641 (PMC6956075; doi:10.3390/microorganisms7120641)
Supplement: Supplementary file 1 [file microorganisms-07-00641-s001.zip › Supplementary_material_S7_relative_species_abundances.docx]

Supplementary Material Table S7: Relative species abundance of SIHUMIx.

Relative species abundances assessed by metaproteomics were determined from day 1 to 15 in SIHUMIx during continuous cultivation of 6 bioreactors.

| day | transit time | *A. caccae* | *B. thetaiotaomicron* | *B. longum* | *B. producta* | *C. butyricum* | *C. ramosum* | *E. coli*  K12 | *L. plantarum* | *Heterogeneous* |
| --- | --- | --- | --- | --- | --- | --- | --- | --- | --- | --- |
| d1 | 12 hTT I | 4.753 | 17.821 | 0.593 | 23.391 | 0.097 | 0.616 | 42.531 | 0.549 | 9.648 |
| d2 | 12 hTT I | 3.812 | 19.403 | 0.468 | 37.251 | 0.175 | 0.864 | 27.228 | 0.201 | 10.598 |
| d3 | 12 hTT I | 3.138 | 40.582 | 0.364 | 29.791 | 0.114 | 0.705 | 14.471 | 0.140 | 10.696 |
| d4 | 12 hTT I | 2.135 | 65.945 | 0.207 | 14.972 | 0.079 | 0.438 | 7.359 | 0.085 | 8.780 |
| d5 | 12 hTT I | 3.180 | 62.451 | 0.836 | 16.998 | 0.033 | 0.696 | 7.748 | 0.223 | 7.836 |
| d6 | 12 hTT I | 2.935 | 64.072 | 0.191 | 16.084 | 0.096 | 0.517 | 7.647 | 0.135 | 8.323 |
| d7 | 12 hTT I | 2.512 | 62.858 | 0.211 | 16.665 | 0.074 | 0.447 | 8.604 | 0.078 | 8.551 |
| d8 | 12 hTT I | 2.435 | 62.882 | 0.266 | 16.677 | 0.094 | 0.403 | 9.088 | 0.082 | 8.073 |
| d9 | 12 hTT I | 2.140 | 66.774 | 0.162 | 13.625 | 0.061 | 0.413 | 9.008 | 0.078 | 7.739 |
| d10 | 12 hTT I | 1.953 | 68.876 | 0.168 | 11.555 | 0.044 | 0.439 | 9.285 | 0.069 | 7.611 |
| d11 | 12 hTT I | 1.552 | 71.635 | 0.138 | 10.548 | 0.025 | 0.504 | 8.161 | 0.108 | 7.329 |
| d12 | 12 hTT I | 1.588 | 70.556 | 0.322 | 10.503 | 0.041 | 0.463 | 8.644 | 0.073 | 7.811 |
| d13 | 12 hTT I | 1.695 | 71.873 | 0.090 | 10.585 | 0.053 | 0.513 | 7.920 | 0.071 | 7.198 |
| d14 | 12 hTT I | 1.840 | 69.087 | 0.306 | 11.270 | 0.041 | 0.507 | 9.029 | 0.069 | 7.851 |
| d15 | 12 hTT I | 1.776 | 70.600 | 0.098 | 11.038 | 0.056 | 0.494 | 8.019 | 0.120 | 7.798 |
| d1 | 12 hTT II | 4.337 | 16.970 | 0.726 | 16.140 | 0.124 | 0.475 | 52.774 | 0.344 | 8.110 |
| d2 | 12 hTT II | 3.586 | 19.343 | 0.597 | 33.971 | 0.214 | 0.832 | 31.227 | 0.195 | 10.036 |
| d3 | 12 hTT II | 4.091 | 24.716 | 0.611 | 32.431 | 0.290 | 0.835 | 26.658 | 0.125 | 10.243 |
| d4 | 12 hTT II | 2.787 | 61.402 | 0.229 | 18.417 | 0.134 | 0.319 | 8.367 | 0.086 | 8.259 |
| d5 | 12 hTT II | 2.412 | 65.340 | 0.178 | 15.420 | 0.063 | 0.350 | 7.473 | 0.102 | 8.661 |
| d6 | 12 hTT II | 2.424 | 63.836 | 0.168 | 16.478 | 0.049 | 0.376 | 7.696 | 0.110 | 8.862 |
| d7 | 12 hTT II | 2.136 | 64.718 | 0.183 | 15.588 | 0.052 | 0.351 | 8.668 | 0.126 | 8.178 |
| d8 | 12 hTT II | 2.004 | 71.577 | 0.182 | 9.880 | 0.028 | 0.445 | 8.736 | 0.139 | 7.010 |
| d9 | 12 hTT II | 2.166 | 66.302 | 0.231 | 13.624 | 0.043 | 0.543 | 9.486 | 0.144 | 7.462 |
| d10 | 12 hTT II | 2.143 | 66.732 | 0.177 | 13.835 | 0.033 | 0.458 | 8.830 | 0.134 | 7.660 |
| d11 | 12 hTT II | 2.120 | 67.370 | 0.231 | 12.917 | 0.042 | 0.453 | 9.269 | 0.157 | 7.441 |
| d12 | 12 hTT II | 2.222 | 67.406 | 0.280 | 11.730 | 0.044 | 0.510 | 10.242 | 0.152 | 7.415 |
| d13 | 12 hTT II | 2.167 | 67.098 | 0.134 | 13.043 | 0.028 | 0.454 | 8.975 | 0.116 | 7.986 |
| d14 | 12 hTT II | 2.357 | 65.860 | 0.137 | 14.654 | 0.030 | 0.487 | 8.444 | 0.104 | 7.926 |
| d15 | 12 hTT II | 2.260 | 66.516 | 0.163 | 13.159 | 0.030 | 0.505 | 9.351 | 0.115 | 7.899 |
| d1 | 24 hTT I | 9.167 | 18.146 | 1.220 | 16.317 | 0.148 | 0.326 | 44.961 | 0.333 | 9.382 |
| d2 | 24 hTT I | 13.280 | 18.548 | 0.784 | 19.516 | 0.176 | 0.290 | 36.994 | 0.319 | 10.093 |
| d3 | 24 hTT I | 14.343 | 19.320 | 0.683 | 27.268 | 0.203 | 0.351 | 26.186 | 0.417 | 11.230 |
| d4 | 24 hTT I | 6.465 | 46.707 | 0.395 | 14.975 | 0.124 | 0.278 | 21.784 | 0.210 | 9.061 |
| d5 | 24 hTT I | 3.062 | 63.724 | 0.241 | 12.007 | 0.094 | 0.256 | 12.065 | 0.109 | 8.443 |
| d6 | 24 hTT I | 3.169 | 62.809 | 0.263 | 13.173 | 0.109 | 0.258 | 11.732 | 0.092 | 8.395 |
| d7 | 24 hTT I | 2.598 | 65.189 | 0.184 | 12.383 | 0.060 | 0.252 | 10.798 | 0.092 | 8.444 |
| d8 | 24 hTT I | 2.827 | 64.227 | 0.186 | 12.365 | 0.058 | 0.254 | 11.462 | 0.093 | 8.529 |
| d9 | 24 hTT I | 2.400 | 71.907 | 0.181 | 7.013 | 0.046 | 0.241 | 10.230 | 0.092 | 7.891 |
| d10 | 24 hTT I | 2.715 | 69.470 | 0.203 | 9.182 | 0.046 | 0.243 | 9.958 | 0.091 | 8.092 |
| d11 | 24 hTT I | 2.632 | 69.378 | 0.178 | 9.509 | 0.041 | 0.238 | 9.986 | 0.095 | 7.943 |
| d12 | 24 hTT I | 2.634 | 68.772 | 0.183 | 10.247 | 0.051 | 0.271 | 9.769 | 0.099 | 7.973 |
| d13 | 24 hTT I | 2.231 | 70.863 | 0.175 | 9.287 | 0.041 | 0.282 | 8.972 | 0.110 | 8.038 |
| d14 | 24 hTT I | 3.105 | 67.829 | 0.208 | 10.053 | 0.078 | 0.276 | 10.366 | 0.089 | 7.996 |
| d15 | 24 hTT I | 4.509 | 66.638 | 0.205 | 9.868 | 0.073 | 0.270 | 10.211 | 0.090 | 8.137 |
| d1 | 24 hTT II | 4.677 | 17.134 | 0.812 | 20.274 | 0.070 | 0.630 | 47.136 | 0.499 | 8.768 |
| d2 | 24 hTT II | 2.944 | 39.217 | 0.413 | 24.812 | 0.087 | 0.635 | 22.543 | 0.248 | 9.101 |
| d3 | 24 hTT II | 1.983 | 68.330 | 0.689 | 11.030 | 0.059 | 0.407 | 8.523 | 0.110 | 8.869 |
| d4 | 24 hTT II | 2.086 | 68.925 | 0.187 | 11.733 | 0.062 | 0.469 | 7.898 | 0.075 | 8.566 |
| d5 | 24 hTT II | 2.282 | 68.300 | 0.139 | 11.917 | 0.050 | 0.547 | 8.523 | 0.075 | 8.167 |
| d6 | 24 hTT II | 2.554 | 66.984 | 0.276 | 12.649 | 0.058 | 0.551 | 8.800 | 0.058 | 8.070 |
| d7 | 24 hTT II | 2.660 | 65.609 | 0.195 | 12.539 | 0.054 | 0.624 | 10.229 | 0.083 | 8.007 |
| d8 | 24 hTT II | 2.502 | 66.314 | 0.234 | 12.918 | 0.057 | 0.598 | 9.583 | 0.058 | 7.735 |
| d9 | 24 hTT II | 2.420 | 68.186 | 0.197 | 11.625 | 0.066 | 0.553 | 9.369 | 0.090 | 7.495 |
| d10 | 24 hTT II | 2.353 | 68.682 | 0.181 | 11.313 | 0.051 | 0.547 | 9.250 | 0.075 | 7.549 |
| d11 | 24 hTT II | 1.865 | 68.077 | 0.282 | 11.825 | 0.038 | 0.594 | 8.854 | 0.064 | 8.400 |
| d12 | 24 hTT II | 1.978 | 70.442 | 0.271 | 9.591 | 0.077 | 0.505 | 9.438 | 0.107 | 7.592 |
| d13 | 24 hTT II | 2.184 | 66.801 | 0.235 | 12.636 | 0.074 | 0.588 | 9.528 | 0.096 | 7.859 |
| d14 | 24 hTT II | 2.283 | 64.667 | 0.241 | 13.307 | 0.057 | 0.605 | 10.944 | 0.096 | 7.802 |
| d15 | 24 hTT II | 2.287 | 65.397 | 0.256 | 13.605 | 0.054 | 0.619 | 9.711 | 0.084 | 7.986 |
| d1 | 48 hTT I | 4.924 | 16.801 | 0.493 | 22.814 | 0.076 | 0.628 | 44.076 | 0.501 | 9.686 |
| d2 | 48 hTT I | 3.368 | 20.604 | 0.350 | 32.487 | 0.137 | 0.956 | 30.823 | 0.240 | 11.034 |
| d3 | 48 hTT I | 1.702 | 65.407 | 0.316 | 15.701 | 0.081 | 0.477 | 7.388 | 0.093 | 8.836 |
| d4 | 48 hTT I | 2.092 | 66.800 | 0.214 | 13.966 | 0.070 | 0.545 | 7.114 | 0.115 | 9.084 |
| d5 | 48 hTT I | 2.297 | 65.387 | 0.219 | 15.186 | 0.081 | 0.589 | 7.429 | 0.109 | 8.704 |
| d6 | 48 hTT I | 2.245 | 65.994 | 0.225 | 14.116 | 0.050 | 0.629 | 8.164 | 0.064 | 8.514 |
| d7 | 48 hTT I | 2.298 | 66.115 | 0.190 | 13.443 | 0.051 | 0.575 | 8.704 | 0.071 | 8.554 |
| d8 | 48 hTT I | 2.226 | 65.666 | 0.166 | 13.184 | 0.052 | 0.538 | 9.752 | 0.062 | 8.354 |
| d9 | 48 hTT I | 2.298 | 65.453 | 0.200 | 13.589 | 0.053 | 0.536 | 9.423 | 0.103 | 8.345 |
| d10 | 48 hTT I | 2.208 | 64.920 | 0.189 | 13.508 | 0.049 | 0.484 | 10.159 | 0.070 | 8.415 |
| d11 | 48 hTT I | 2.185 | 65.521 | 0.416 | 12.540 | 0.099 | 0.639 | 10.398 | 0.088 | 8.113 |
| d12 | 48 hTT I | 1.949 | 66.309 | 0.305 | 13.748 | 0.049 | 0.513 | 8.683 | 0.067 | 8.378 |
| d13 | 48 hTT I | 1.930 | 66.154 | 0.302 | 13.693 | 0.045 | 0.545 | 8.801 | 0.068 | 8.461 |
| d14 | 48 hTT I | 1.983 | 66.703 | 0.272 | 13.159 | 0.044 | 0.546 | 8.827 | 0.072 | 8.393 |
| d15 | 48 hTT I | 1.884 | 66.898 | 0.178 | 12.921 | 0.042 | 0.575 | 8.936 | 0.072 | 8.494 |
| d1 | 48 hTT II | 8.625 | 20.016 | 1.275 | 12.757 | 0.163 | 0.334 | 47.918 | 0.345 | 8.567 |
| d2 | 48 hTT II | 12.368 | 21.200 | 1.016 | 21.789 | 0.247 | 0.370 | 32.349 | 0.415 | 10.247 |
| d3 | 48 hTT II | 14.463 | 23.435 | 0.761 | 25.821 | 0.273 | 0.449 | 23.411 | 0.429 | 10.958 |
| d4 | 48 hTT II | 14.351 | 24.297 | 0.788 | 24.447 | 0.283 | 0.468 | 24.195 | 0.374 | 10.797 |
| d5 | 48 hTT II | 7.316 | 53.508 | 0.376 | 13.042 | 0.167 | 0.398 | 16.407 | 0.193 | 8.594 |
| d6 | 48 hTT II | 4.804 | 62.176 | 0.379 | 12.422 | 0.116 | 0.368 | 11.185 | 0.176 | 8.374 |
| d7 | 48 hTT II | 4.579 | 62.685 | 0.259 | 12.361 | 0.095 | 0.313 | 10.972 | 0.119 | 8.618 |
| d8 | 48 hTT II | 4.236 | 63.802 | 0.238 | 11.484 | 0.093 | 0.288 | 11.427 | 0.117 | 8.315 |
| d9 | 48 hTT II | 4.263 | 63.033 | 0.240 | 12.589 | 0.109 | 0.324 | 10.919 | 0.115 | 8.408 |
| d10 | 48 hTT II | 4.015 | 64.598 | 0.295 | 11.098 | 0.115 | 0.287 | 11.281 | 0.108 | 8.202 |
| d11 | 48 hTT II | 3.788 | 63.412 | 0.313 | 12.734 | 0.151 | 0.320 | 10.902 | 0.131 | 8.248 |
| d12 | 48 hTT II | 2.996 | 64.719 | 0.223 | 12.598 | 0.063 | 0.375 | 10.800 | 0.130 | 8.095 |
| d13 | 48 hTT II | 2.970 | 66.716 | 0.240 | 10.880 | 0.108 | 0.348 | 10.698 | 0.126 | 7.915 |
| d14 | 48 hTT II | 3.300 | 65.022 | 0.220 | 12.260 | 0.098 | 0.338 | 10.529 | 0.138 | 8.095 |
| d15 | 48 hTT II | 2.815 | 67.081 | 0.161 | 11.648 | 0.097 | 0.292 | 9.821 | 0.101 | 7.984 |
